# Nonsteroidal anti-inflammatory drug use in patients with chronic kidney disease

**DOI:** 10.1007/s40620-016-0352-z

**Published:** 2016-09-27

**Authors:** Zbigniew Heleniak, Magdalena Cieplińska, Tomasz Szychliński, Dymitr Rychter, Kalina Jagodzińska, Alicja Kłos, Izabela Kuźmiuk, Marzena Jakimowicz Tylicka, Leszek Tylicki, Bolesław Rutkowski, Alicja Dębska-Ślizień

**Affiliations:** 1 Department of Nephrology, Transplantology and Internal Medicine, Medical University of Gdańsk, Gdańsk, Poland; 2 Unit of Clinical Pharmacology in the Department of Nephrology, Transplantology and Internal Medicine, Medical University of Gdańsk, 7 Dębinki Street, 80-952 Gdańsk, Poland

**Keywords:** Chronic kidney disease, NSAIDs, Pain, Side effects

## Abstract

**Aims:**

Nonsteroidal anti-inflammatory drugs (NSAIDs) are the cornerstone of pain management. There are no detailed data on NSAIDs use in Poland, especially in patients with chronic kidney disease (CKD). The aim of this study was to evaluate the frequency, circumstances, and causes of NSAIDs use as well as knowledge of their side-effects in patients with CKD.

**Method:**

This cross-sectional study was conducted in 972 individuals with CKD, enrolled in a written survey originally developed by the authors. There were 574 patients with CKD stage I-IV, 414 patients after renal transplantation stage II-IV (CKDT) and 84 dialyzed patients (44 peritoneal, 40 hemodialysis).

**Results:**

Among the entire study group, 16.9 % of patients used NSAIDs every day, or several times a week. The average number of tablets taken within a month was 21.8. Subgroup analysis revealed that NSAIDs were taken most often by patients on hemodialysis: 35 % of them used NSAIDs every day or several times a week (43.15 pills per month). The most common reason for using NSAIDs were bone-joint pain (29.3 %) and headache (26.2 %). Side effects of painkillers such as renal function deterioration and the possible promotion of stomach ulcers were experienced by 43.6 and 37.6 % of respondents, respectively.

**Conclusion:**

Patients with CKD often take NSAIDs. This applies especially to the group of people undergoing hemodialysis, which is mainly associated with chronic osteo-articular pain. The results also show a low awareness of painkillers’ adverse effects.

## Introduction

Almost 40 % of patients visit primary care providers due to mild to moderate acute pain [1]. Moreover, about 70 % of emergency departments’ interventions are associated with pain complaints [2]. In Poland, 24 % of the general population, in the case of discomfort or pain, prefer self-treatment using pharmaceuticals, including non-steroid anti-inflammatory drugs (NSAIDs), to the doctor’s advice [2]. NSAIDs are mostly available in Poland without prescription (over the counter, OTC). Reports show also that Poland is in the 6th position in Europe in terms of OTC sales of painkillers [3].

NSAIDs are the cornerstone of pain management in patients who have inflammatory pain, acute pain (e.g. headache, postoperative pain, and orthopedic fractures) or chronic pain (e.g. rheumatoid arthritis, osteoarthritis, and gout) [4]. Approximately 70 % of people aged over 65 years use NSAIDs at least once a week, and half of them take at least 7 doses per week. Both traditional NSAIDs, and the second generation cyclooxygenase-2 inhibitors offer superior efficacy compared with acetaminophen, but they also carry a significant risk for serious gastrointestinal, cardiovascular and renal adverse events [5, 6]. NSAIDs use has been associated with both acute kidney injury in the general population, and chronic kidney disease (CKD) progression in those with chronic nephropathies [7]. In addition, NSAIDs interact unfavorably with some commonly prescribed medications in CKD patients, including loop diuretics, angiotensin-converting enzyme inhibitors, and angiotensin receptor blockers, leading to their reduced effectiveness along with an increased risk of renal impairment [8].

Despite these potential adverse effects of NSAIDs in CKD patients, little is known about the patterns of NSAIDs use in this population. The aim of this study was to evaluate the frequency, circumstances, and causes of NSAIDs’ use, as well as knowledge of their side-effects in patients with CKD.

## Materials and Methods

A cross-sectional survey study was conducted in the first 6 months of 2014 in patients with CKD who were under the care of the Department of Nephrology, Medical University of Gdansk, Poland. This survey was offered to all 1,300 patients who were under the care of the local Outpatient Unit, and the Hemodialysis and Peritoneal Dialysis Units. The written survey was originally developed by the authors. It consisted of 22 questions. Aspirin used in small doses (i.e. 75 mg) was excluded from our primary definitions of NSAIDs because of its relatively low adverse effects rate, and because its use is commonly recommended for the prevention of comorbid cardiovascular complications. To anyone having difficulty completing the written survey, support was given from students of the Medical Faculty, Medical University of Gdansk.

Data was evaluated using a STATISTICA (version 12.0 Stat Soft Inc.) software package. The results are presented as mean value ± standard deviation (SD), median, interquartile range and numbers or percent frequency, as appropriate. The quantitative variable differences were assessed by analysis of variance (ANOVA) or the non-parametric Kruskal–Wallis test. In order to measure the differences in prevalence between selected categories, we used the Chi square (χ^2^) test for the contingency tables. A p < 0.05 (two-tailed) was considered statistically significant.

## Results

A total of 972 individuals (74.8 %) responded to the survey. There were 574 patients with CKD stage I-IV (all patients with CKD stage V were under renal replacement therapy), 314 patients after kidney transplantation (CKDT), and 84 dialyzed patients: 44 peritoneal (PD) and 40 hemodialysis (HD). Hypertension, cardiovascular and bone-joint diseases were the most frequent comorbidities of our study population, and occurred in 79, 27.4 and 21.5 % respectively. Detailed characteristics of the group are presented in Tables 1 and 2. Hemodialysis patients were the oldest compared to the other subgroups (ANOVA: p < 0.001).

**Table 1:** Characteristics of the study population

Number of patients	972
M	464 (47.7 %)
CKD 1–4	574 (59.1 %)
Peritoneal dialysis	44 (4.5 %)
Hemodialysis	40 (4.1 %)
Kidney transplantation	314 (32.3 %)
Age (years)	
Average ± SD	55.0 ± 17.5
Duration of CKD from diagnosis (years)	
Median and IQR	10; 5–20
BMI (kg/m2)	
Average ± SD	26.3 ± 5.0
Education	
Basic/technical	290 (29.8 %)
Secondary	420 (43.2 %)
Higher	262 (27.0 %)
Other diseases	
Hypertension	768 (79.0 %)
Diabetes	226 (23.2 %)
Cardiovascular disease	266 (27.4 %)
Cancer	78 (8.0 %)
Bone and joint disease	209 (21.5 %)
Average number of drugs	
Median and IQR	5.5; 3.0–8.0

*IQR* interquartile range, *SD* standard deviation, *BMI* body mass index, *CKD* chronic kidney disease

**Table 2:** Characteristics of the study subgroups

	CKD 1–4	Kidney transplantation (CKDT)	Hemodialysis (HD)	Peritoneal dialysis (PD)
Age (years)				
Average* ± SD	57.2 ± 19.4	50.1 ± 13.6	61.3 ± 11.1	55.9 ± 14.5
Gender M/F	292/282	178/136	17/23	21/23
BMI (kg/m^2^)				
Average ± SD	26.8 ± 5.4	25.6 ± 4.1	24.1 ± 3.8	26.1 ± 4.4
Duration of CKD from diagnosis (years)				
Median and IQR	7.0; 3.0–15.0	15.0; 10.0–22.0	14.0; 7.0–28.0	5.0; 3.0-14.5
Bone-joint disease, n (%)**	153 (27.5)	42 (13.4)	21 (52.5)	2 (4.5)
Cancer, n (%)**	60 (10.4)	13 (4.1)	1 (2.5)	4 (9.1)
Diabetes, n (%)**	135 (23.5)	70 (22.3)	10 (25.0)	11 (25.0)
Hypertension, n (%)**	432 (75.1)	267 (85.0)	30 (75.0)	40 (91.0)
Cardiovascular disease, n (%)**	175 (30.5)	59 (18.8)	18 (45.0)	14 (31.8)

*IQR* interquartile range, *CKD* chronic kidney disease

*ANOVA p < 0.001 (difference between study subgroups: CKD 1–4 vs. CKDT vs. HD vs. PD)

**χ^2^ test p < 0.001 (difference between study subgroups: CKD 1–4 vs. CKDT vs. HD vs. PD)

### NSAIDs use

**Fig. 1:**
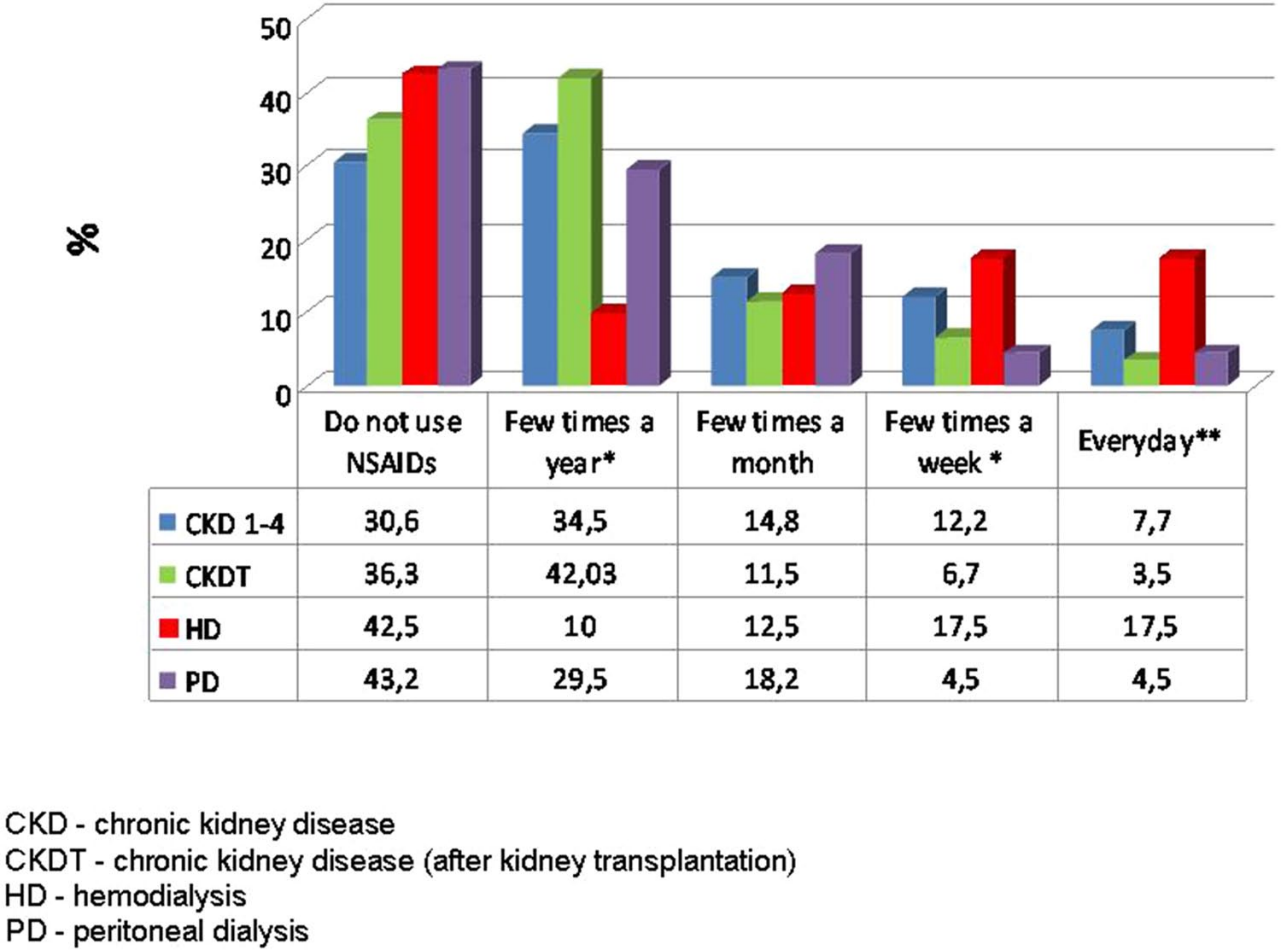
The frequency of use of NSAIDs in subgroups. *χ^2^ test p < 0.01 (difference between study subgroups: CKD 1–4 vs. CKDT vs. HD vs. PD). **χ^2^ test p < 0.001 (difference between study subgroups: CKD 1–4 vs. CKDT vs. HD vs. PD)

**Fig. 2:**
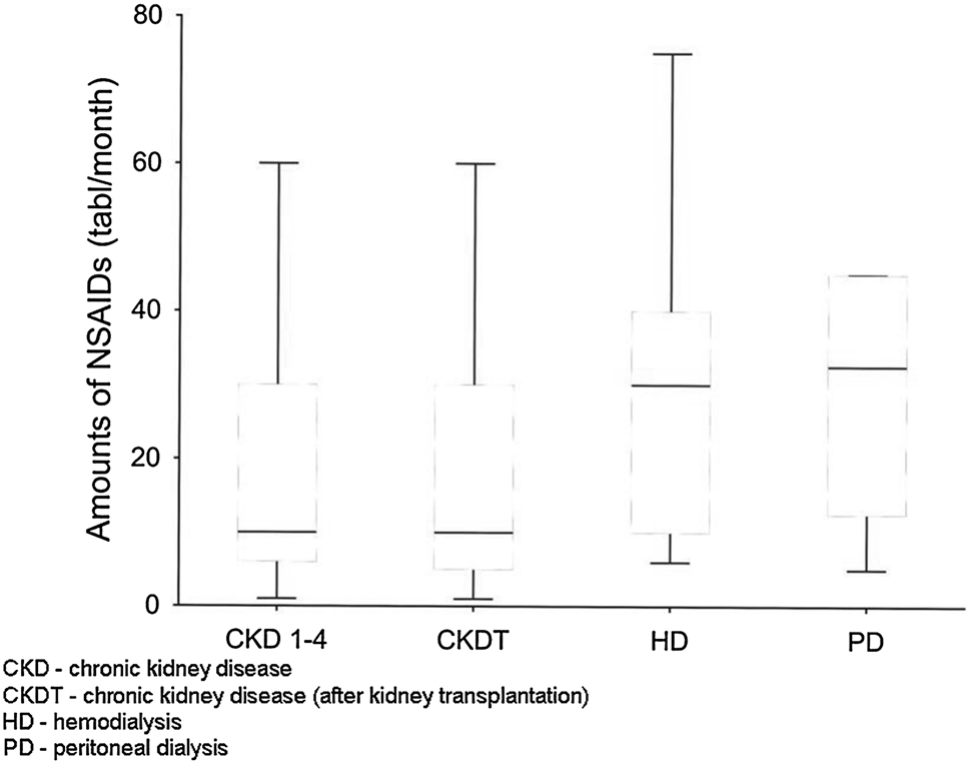
The median and I, II quartyl of the number of NSAIDs tablets taken per month by patients who used NSAIDs regularly i.e. every day or a few times a week

Among the entire study group, as many as 6.58 % of patients used NSAIDs every day, 10.3 % a few times a week, and 13.8 % a few times a month; 35.7 % of them took NSAIDs occasionally, i.e. a few times a year, while 33.5 % reported not to use NSAIDs. The median number of tablets taken within a month, in persons using NSAIDs regularly, i.e. every day or a few times a week, was 20. Use of two NSAIDs at the same time was reported by 13 % of respondents. The most common reasons for using NSAIDs were bone-joint pain (29.3 %), and headache (26.2 %) (Table 3). The decision about taking NSAIDs was made in 46.71 % of cases by the patients themselves, in 43.41 % by the doctor, and in 9.88 % by a pharmacist. Subgroup analyses revealed that NSAIDs were taken most often by hemodialysis patients: 17.5 % of them took pills every day, and 17.5 % used NSAIDs a few times a week (Fig. 1). The median number of NSAIDs taken within a month in the hemodialysis and peritoneal dialysis patients who used NSAIDs regularly, i.e. every day or a few times a week, was 30 and 32.5 tablets, respectively (Fig. 2).

**Table 3:** Causes of use of NSAIDs (%)

Cause (%)	Study population	CKD 1–4	CKDT	HD	PD
Abdominal pain	7.7	5.2	7.1	10.3	11.1
Headache	26.2	22.4	34.7	31.0	29.6
Menstrual cramp	6.9	6.8	2.9	2.0	3.7
Bone and joint pain	29.3	25.6	19.7	69.0	33.3
Inflammation	15.5	12.1	18.2	15.4	22.2
Other	14.4	14.1	11.8	16.3	13.2

*CKD* chronic kidney disease, *CKDT* chronic kidney disease after kidney transplantation, *HD* hemodialysis, *PD* peritoneal dialysis

### Side-effects knowledge

**Fig. 3:**
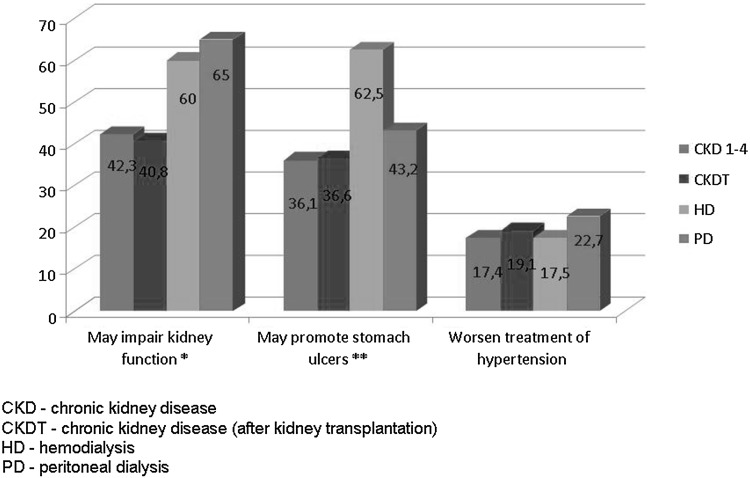
The knowledge of the adverse effects of analgesics in subgroups. *χ^2^ test p < 0.01 (difference between study subgroups: CKD 1–4 vs. CKDT vs. HD vs. PD). **χ^2^ test p < 0.001 (difference between study subgroups: CKD 1–4 vs. CKDT vs. HD vs. PD)

As many as 54.1 % of the respondents using NSAIDs did not take into account their side-effects. The most common side-effects of NSAIDs such as a possible deterioration of renal function, peptic ulcer disease (PUD), and impairment of blood pressure control were known by 43.6, 37.6 and 18.2 % of respondents, respectively. The patients who themselves decided to use NSAIDs derived their knowledge about these drugs from the media (72.5 %), friends (15.0%) and pharmacists (12.5 %). Subgroup analysis revealed that knowledge about potential deterioration of renal function by NSAIDs was greatest among PD patients (χ^2^ test: p < 0.01), while knowledge about promotion of stomach ulcers was greatest among HD patients (χ^2^ test: p < 0.05) (Fig. 3).

## Discussion

CKD patients seem to be particularly vulnerable to the occurrence of the side-effects of NSAIDs, in particular, to the adverse effects of NSAIDs on blood pressure control, decreased renal blood flow, and promotion of PUD [7]. The prevalence of hypertension may be as high as 90 % in the CKD population. Through a variety of mechanisms related to prostaglandin inhibition, including sodium retention and vasoconstriction, NSAIDs may increase blood pressure. NSAIDs have been shown to produce a clinically significant increment in mean blood pressure by 5 mmHg, most marked in patients with controlled hypertension [9]. The incidence of PUD in patients with CKD is 10 to 12 times higher than in patients without CKD. In particular, PUD affects chronically hemodialyzed persons. One of the most important risk factors for the development of PUD and its complications is the use of NSAIDs [9]. NSAIDs use is also associated with a threefold greater risk for acute renal failure compared to non-NSAIDs use, after adjusting for age, sex, body mass index, and several comorbidities [6]. In addition, NSAIDs may cause deterioration in renal function in patients with CKD especially in those treated with renin angiotensin system blockers, or with episodes of hypotension [10, 11]. This may be particularly dangerous for renal transplant recipients, or patients with stage CKD II-IV. Given all these facts, it is not surprising that knowledge concerning the use of NSAIDs in CKD patients is of great importance.

The study showed that approximately 17 % of patients with CKD, and more than one-third of the HD patients, use NSAIDs daily or several times a week. Among them, up to 13 % of patients reported using two different drugs at the same time. The percentage of PD patients taking NSAIDs regularly is low. Nevertheless, those who take medications regularly use them in large quantities comparable to the number of tablets used by HD patients. This, despite the fact that the awareness of the side-effects of NSAIDs is relatively large in patients treated with peritoneal dialysis.

These data seem to be a real cause for concern. The current use of NSAIDs, particularly those available OTC, is lower in the US CKD population, in whom approximately 5 % of patients with moderate to severe CKD took NSAIDs regularly [12]. In the North West Adelaide Health Study (NWAHS) in 2004–2006, a longitudinal representative population study from Australia, 16 % of patients with CKD were using NSAIDs regularly [13]. NSAIDs were highly prescribed in CKD patients from the general population of Southern Italy. The general practice “Arianna” database containing data from 158,510 persons, registered with 123 general practitioners (GPs) showed that 35.6 % of CKD patients were treated with NSAIDs for periods exceeding 90 days [14].

The most common reason for starting therapy with NSAIDs in CKD patients is the occurrence of osteoarthrosis, rheumatoid arthritis, CKD-mineral and bone disorders, or arthralgia [10, 17]. Pain, as an everyday complaint, is reported to be a very common problem in these comorbidities. Pain may be also a direct result of the hemodialysis procedure, and is reported by 40–50 % of HD patients [15]. It refers to muscle cramps, pain related to needles and catheters used in dialysis, painful syndromes such as calciphylaxis, nephrogenic sclerosing fibrosis, and dialysis-related amyloidosis [16]. The results of our study are consistent with these facts. Bone-joint diseases were very frequent among the study respondents, and occurred in 21.5 % of the entire population, and in as many as 52.5 % of HD patients. They were also the most common reason for NSAIDs use in our study.

The wide and easy availability of OTC medicines has led to an uncritical use of these agents in Poland, where 52 % of the general population consider that the use of OTC drugs including NSAIDs is safe [3]. In 46 % of cases, people before taking these agents do not read the accompanying leaflet, or do so only sporadically [3]. Half of them choose a drug based only on advertising in the media, or on the opinion of family and friends [2]. The results of our study indicate that the situation is similar also in CKD patients. The decision about NSAIDs is taken in 46.7 % of cases by the patients themselves, and as many as 54.1 % do not reflect on their possible side-effects. Simultaneously, the knowledge about the side-effects of NSAIDs is not very high, ranging from 18 to 46 % depending on the specific issue.

Interestingly, awareness of the side-effects of NSAIDs is greatest among patients undergoing hemodialysis. Despite this, the consumption of NSAIDs is the highest in this group. It may indicate that bone-disease problems are a very important, and still unsolved, issue. Of great concern, awareness about the risks arising from the use of NSAIDs is particularly poor in patients after renal transplantation. Only 19 and 40 % of them were aware that use of NSAIDs could lead to a possible deterioration of blood pressure control or of graft function, respectively. Given these facts, specific education is urgently required in this population. Intervention strategies to improve the prescribing pattern in CKD patients should be also targeted to GPs and pharmacists, who often recommend NSAIDs in this specific population [14].

## Conclusions

Patients with CKD quite often take NSAIDs. This applies especially to patients undergoing hemodialysis, where NSAID use is mainly associated with their chronic osteo-articular complaints. In addition, our findings show a low awareness of the adverse side-effects NSIADs, which is especially alarming in patients after renal transplantation.
